# JDP2, a Novel Molecular Key in Heart Failure and Atrial Fibrillation?

**DOI:** 10.3390/ijms22084110

**Published:** 2021-04-16

**Authors:** Gerhild Euler, Jens Kockskämper, Rainer Schulz, Mariana S. Parahuleva

**Affiliations:** 1 Institute of Physiology, Justus Liebig University, 35392 Giessen, Germany; Rainer.schulz@physiologie.med.uni-giessen.de; 2 Biochemical-Pharmacological Centre (BPC) Marburg, Institute of Pharmacology and Clinical Pharmacy, University of Marburg, 35043 Marburg, Germany; jens.kockskaemper@staff.uni-marburg.de; 3 Internal Medicine/Cardiology and Angiology, University Hospital of Giessen and Marburg, 35033 Marburg, Germany; mariana.parahuleva@prof-parahuleva.de

**Keywords:** heart failure, atrial fibrillation, transcription factor, remodeling

## Abstract

Heart failure (HF) and atrial fibrillation (AF) are two major life-threatening diseases worldwide. Causes and mechanisms are incompletely understood, yet current therapies are unable to stop disease progression. In this review, we focus on the contribution of the transcriptional modulator, Jun dimerization protein 2 (JDP2), and on HF and AF development. In recent years, JDP2 has been identified as a potential prognostic marker for HF development after myocardial infarction. This close correlation to the disease development suggests that JDP2 may be involved in initiation and progression of HF as well as in cardiac dysfunction. Although no studies have been done in *humans* yet, studies on genetically modified *mice* impressively show involvement of JDP2 in HF and AF, making it an interesting therapeutic target.

## 1. Introduction

Despite the progress in medical therapies, the incidence of heart failure (HF) is still rising. From 1990 until 2017, the number of patients with HF worldwide nearly doubled [1]. Myocardial infarction (MI) and hypertension are among the main causes of HF. Similarly, atrial fibrillation (AF) is a life-threatening cardiac disease with increasing prevalence, in particular in industrialized countries with an aging population [2,3]. HF and AF share common risk factors. There is a mutual relationship between HF and AF, with HF increasing the risk to develop AF and vice versa [2,3]. How to stop the progress from the initial events to HF or AF is still unclear, and the need to discover new targets from which new therapeutic approaches can be derived is great. In this review, we focus on the transcription modulating factor Jun dimerization protein 2 (JDP2), because some recent studies revealed quite strong correlations between increased JDP2 levels and HF progression or development of atrial arrhythmias. By bringing together findings from animal studies on the action of JDP2 in the cardiovascular system, we provide evidence that induction of JDP2 may also be causally involved in the development of both HF and AF in patients.

## 2. Transcriptional Control by *JDP2*

The field on transcriptional control mechanisms by JDP2 was reviewed in detail by Tsai et al. [4]. Therefore, a short overview about the main control points of JDP2 will be given in this chapter. JDP2 belongs to the leucine zipper superfamily of transcription factors that predominantly binds to the promoter elements cAMP response element (CRE) or 12-O-tetradecanoylphorbol-13-acetate (TPA) response element (TRE) [5,6]. It was originally described as a transcriptional repressor of activator protein 1 (AP-1). JDP2 displaces the typical binding partner from the AP-1 dimer, thereby producing a transcriptionally inactive complex.

Meanwhile, knowledge about the influence of JDP2 on transcription has dramatically increased. It became clear that JDP2 not only represses transcription via the AP-1 blockade, but also via binding to core histones and nucleosomes in a sequence specific manner. In this situation, JDP2 provokes chromatin remodeling, since it prevents histone modifying and transcription promoting enzymes, such as histone acetyltransferases (HAT) or methylases, from accessing the nucleosome [7,8]. In addition, JDP2 can recruit histone deacetylases into the complex as a further regulating step in transcriptional inhibition [9,10,11]. Besides these pleiotropic effects on transcriptional repression, JDP2 can also activate transcription. It acts as a coactivator of the progesterone receptor [12,13,14,15]. By binding to anti-oxidant responsive element (ARE) sites, in association with the Nrf2/MafK complex, JDP2 promotes the transcription of antioxidant genes [16,17]. Thus, overall it can be said, that JDP2 primarily acts as a sequence specific inhibitor of transcription, but in some instances it may also promote transcription.

## 3. Correlation between JDP2 Expression and Heart Failure

**Figure 1:**
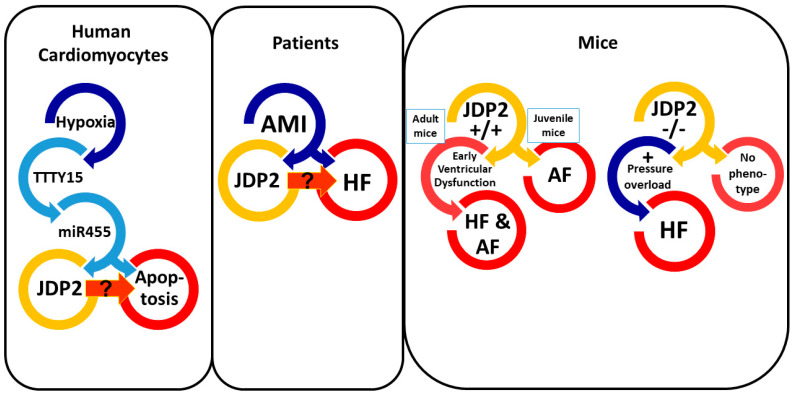
Overview about cardiac JDP2 effects. Summarized are results of studies about JDP2 effects in the heart and isolated cardiomyocytes.

The first report about the association of increased JDP2 expression after myocardial infarction (MI) with HF progression was published by Maciejak and coworkers in 2015 [18]. They collected peripheral blood samples from patients with acute myocardial infarction (AMI) at admission and at three consecutive dates following admission, with the latest 6 months after AMI. Gene profiling in peripheral blood mononuclear cells (PBMCs) of these patients revealed differential gene expression at admission in patient groups that developed HF within 6 months after AMI compared to the patient group who did not. *JDP2* was among these differentially expressed genes with an upregulation at admission and up to 6 days after AMI. Thus, JDP2 expression in AMI patients indicates a more severe initial damage to the heart, which then culminates in HF at later time points. The analysis of the predictive value of JDP2 as a prognostic marker for HF revealed at a cut-off value of 1.7-fold change 88.9% sensitivity and 87.5% specificity. This indicates JDP2 may be a valuable biomarker for prediction of HF in AMI patients (Figure 1 and Table 1). In another study, Qui and Liu [19] analyzed two independent datasets from gene expression studies of peripheral blood cells from MI patients, and identified 477 conserved genes that were differentially expressed in both datasets, and *JDP2* was one of them. Functional enrichment analysis and biological network analysis of the conserved genes revealed several key genes, such as *MAPK14*, *STAT3*, and *MAPKAPK2*, with *JDP2* being a central part in the protein-protein interaction network in MI patients. The latest study revealing JDP2 as a prognostic marker for HF development was just recently published [20]. The aim of the study by Wang and Cao was to find valuable microRNAs (miRNAs/miRs) and target mRNAs in blood of MI patients. Therefore, one miRNA dataset and four mRNA datasets were selected for integrated analysis. Within 1007 differentially expressed mRNAs, *JDP2* belonged to the top 20 differentially expressed mRNAs. Several regulatory interaction pairs between miRNA and mRNA were identified. *JDP2* was identified as target of hsa-mir-17-3p. These results were validated in a small group of MI patients and confirmed downregulation of hsa-mir-17-3p and upregulation of *JDP2* mRNA in blood of patients with AMI. Thereafter, *JDP2* was significantly downregulated at discharge compared with that at admission. Interestingly, upregulation of hsa-mir-17-3p was demonstrated to promote the viability of cardiomyocytes [21]. Thus, the identification of hsa-mir-17-3p downregulation and *JDP2* upregulation in blood in AMI patients indicates a possible role of this miR-17-3p/mRNA pair in the impairment of cardiomyocyte viability that may contribute to HF development.

Most studies in *humans*, which are related to JDP2 expression in MI patients, were conducted on peripheral blood samples. Whether the peripheral JDP2 upregulation is also reflected in cardiac tissue has not been rigorously evaluated yet. Just recently, upregulation of long noncoding RNA TTTY15 has been found in MI and in *human* cardiomyocytes under hypoxia [22]. TTTY15 targets miR-455, which regulates JDP2 expression. Thus, there is evidence of JDP2 induction under hypoxic conditions in *human* cardiomyocytes (Figure 1).

## 4. Influence of JDP2 on Ventricular Remodeling and Function

Due to the strong correlations of JDP2 expression after MI in *humans* with the progression to HF, there is great interest to elucidate, if JDP2 contributes to development of HF. Transgenic *mice* with JDP2 overexpression or *JDP2 knockout* (KO) offer a great opportunity to find answers to this question. Kehat and colleagues, therefore, established transgenic *mice* with JDP2 overexpression, specifically in the heart, in a temporally controlled manner [23]. These *mice* were born at an expected ratio; however, 4-week-old *mice* displayed a 22% higher mortality compared to wild type (WT). At that age, a slight ventricular hypertrophy was apparent. Echocardiography did not reveal any increase in ventricular wall or volume dimensions nor any impairment of ventricular function. However, atria of these *mice* revealed massive atrial dilatation and, as discussed later in detail, impairments in atrial function seemed to be the prevailing cause of the increased mortality of four-week-old *JDP2 mice* [23].

**Figure 2:**
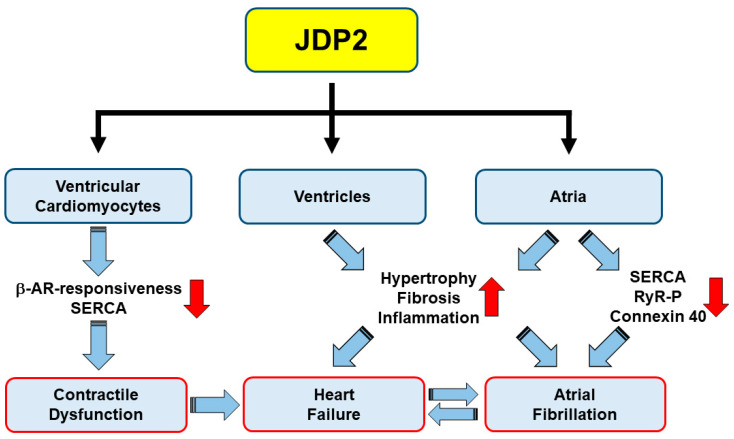
Potential mechanisms contributing to heart failure and atrial fibrillation in *JDP2 mice*.

Since JDP2 was expressed in this study during juvenile development, another study set out to determine effects of JDP2 overexpression in adult *mice* [24]. Because *JDP2* expression was under control of a tetracycline-sensitive promoter, feeding of mothers and newborn animals with doxycycline up to 4 weeks enabled the repression of *JDP2* transcription during juvenile development. Overexpression of JDP2 for one week in adult *mice* provoked ventricular dysfunction, since cardiac output, fractional shortening, and ejection fraction declined. This ventricular dysfunction aggravated during prolongation of JDP2 overexpression up to five weeks. Contractile dysfunction was observed also on a cellular level. Isolation of ventricular cardiomyocytes from these *mice* revealed reduced cell shortening and contraction velocity under electrical stimulation after one week of JDP2 overexpression. After prolonged JDP2 overexpression, the contractile capacity declined further, and after lifelong JDP2 overexpression, positive inotropic effects by beta-adrenergic stimulation were abolished [26] (Figure 2). This indicates that the contractile ventricular dysfunction in vivo is based, at least in part, on the cardiomyocytes themselves. This assumption is supported by the reduction of the calcium pump SERCA in ventricles of JDP2 overexpressing adult *mice* [24]. Both impaired beta-adrenergic responsiveness and reduced expression of SERCA are hallmarks of *human* HF [29].

Furthermore, ventricular remodeling was evident in these *mice*, demonstrated by increases in hypertrophy and fibrosis after 5 weeks of JDP2 overexpression. This structural remodeling imposes an additional burden on the ventricle and may aggravate ventricular dysfunction. Interestingly, the finding on hypertrophy induction in *JDP2 mice* could not be confirmed in isolated cardiomyocytes. On the contrary, ventricular cardiomyocytes of *JDP2 mice* were even protected against hypertrophic growth stimulation [26]. Whereas in the cell culture system, only one pro-hypertrophic stimulus was tested, in the in vivo heart, many factors may impose stress on the ventricle and, thereby, override the defense system, and finally provoke ventricular hypertrophy. Moreover, as hypertrophy was determined by heart weight to body weight ratio, and not by cardiomyocyte size in histology sections, the increase in HW/BW may be provoked mainly by the ventricular fibrosis.

Another interesting finding in *JDP2 mice* is the upregulation of pro-inflammatory marker gene expression in the ventricles. Since enhanced JDP2 expression in *humans* was analyzed only in peripheral blood cells, it is intriguing as to whether peripheral blood cells are attracted into the hearts of *JDP2 mice* and thereby contribute to ventricular dysfunction. *Mice* overexpressing the JDP2 homolog ATF3 also develop cardiac hypertrophy and ventricular dysfunction. Macrophage-cardiomyocyte crosstalk plays a central role in adverse cardiac remodeling by ATF3, since either heart-specific or macrophage-specific *ATF3* ablation blunts the hypertrophic response to chronic pressure overload [30]. Thus, under pressure overload, cardiomyocytes induce an inflammatory response via ATF3, leading to macrophage recruitment to the heart and maladaptive cardiac remodeling. It remains to be elucidated, if this holds true also for *JDP2 mice*; but the close homology between JDP2 and ATF3 makes this hypothesis quite likely. This macrophage-cardiomyocyte crosstalk also indicates that the increased JDP2 expression levels found in peripheral blood of MI patients may contribute to HF progression.

Given the fact that JDP2 overexpression provokes cardiac dysfunction, one might assume that *JDP2 KO* protects against HF induction. Surprisingly, *JDP2 KO* did not protect the heart against pressure overload induced damage following transverse aortic constriction (TAC). Instead, the *JDP2 KO mice* performed worse than wild type [27]. Thus, both, JDP2 overexpression and *JDP2* deletion are related to ventricular dysfunction (Figure 1). However, there are obvious differences that may explain the unexpected outcome. First, whereas JDP2 overexpression was restricted to heart tissue due to the control under alpha-MHC promoter, the KO was a global deletion of *JDP2*, which might bear unexpected side effects. Second, under JDP2 overexpression ventricular dysfunction developed spontaneously, whereas *JDP2 KO mice* primarily presented no obvious phenotype. Only in response to pressure overload the absence of JDP2 caused stronger ventricular impairments compared to *WT mice*. However, hypertrophic enlargement of the heart was already detectable in *JDP2 KO mice* without any additional provocation, indicating that basal JDP2 expression is responsible for the control of cardiac size and function in the healthy heart. This fits quite well to the findings that isolated ventricular cardiomyocytes are protected against induction of hypertrophic growth in vitro [26]. However, when the heart is exposed to sustained stress, i.e., under pressure overload or JDP2 overexpression, contractile impairment of cardiomyocytes assumes the prevailing role and HF progresses.

Interestingly, the severe cardiac phenotype of *JDP2 KO mice* under pressure overload was ameliorated *in JDP2/ATF3 double KO mice* with less fibrosis, reduced inflammatory, and hypertrophic gene expression, and preserved cardiac function [28]. Via an ATF/CRE binding site in the promoter of the *ATF3* gene, JDP2 can repress *ATF3* transcription [31], and ATF3 levels were indeed increased in *JDP2 KO mice* [27]. Transgenic *mice* with cardiac overexpression of ATF3 display maladaptive cardiac remodeling and reduced cardiac function [32,33]. Thus, under stress, JDP2 can act as an ATF3 repressor that prevents induction of ATF3 and, thereby, protects against HF development. However, when JDP2 itself gets overexpressed, it also reveals detrimental functions, similar to ATF3. This is not surprising since ATF3 and JDP2 are close structural homologues with similar target sequences in the promoter of diverse genes, which almost automatically should produce similar results when they are activated. Thus, the balance of JDP2 and ATF3 seems to be a critical factor for HF development. Low levels of both are predictors of adaptive/protective remodeling processes, but enhanced and prolonged expression will be detrimental.

## 5. JDP2 Promotes Atrial Remodeling and Arrhythmias

Much clearer, and without any doubt, is the influence of JDP2 on the induction of atrial arrhythmias, such as conduction defects or AF. Already the first description of transgenic *JDP2 mice* with a continuous cardio-specific JDP2 overexpression from birth up to 4 weeks of age characterized JDP2 as a major mediator of massive bi-atrial dilatation [23]. ECG recordings on anesthetized animals revealed conduction abnormalities and occurrence of AF (Figure 1). The observed connexin 40 downregulation under JDP2 overexpression may contribute to prolongation of atrial conduction times, since the electrical coupling is reduced in *connexin KO mice* [34]. Interestingly, arrhythmias and atrial dilatation were almost fully reversible upon cessation of JDP2 overexpression [23]. The absence of ventricular impairments at that time point under JDP2 expression in juvenile *mice* suggested development of atrial defects without any secondary effects on ventricles.

Just recently, we extended these studies on *JDP2 mice*, starting with JDP2 overexpression in adult *mice* at the age of 5 weeks. ECGs were recorded on non-anesthetized *mice* in order to exclude side effects of anesthetics on heart rhythm [25]. Within 4 to 5 weeks of JDP2 overexpression, atrial dilatation and fibrosis, prolongation of conduction times, and episodes of AF became evident (Figure 1). Within this time, reduced expression and phosphorylation of calcium handling proteins (SERCA, RyR2) and connexin 40, as well as a massive increase in pro-inflammatory marker genes, such as *MCP1*, was detected. Therefore, dysregulated calcium handling and reduced electrical coupling of atrial myocytes likely contribute to atrial dysfunction (Figure 2). Moreover, increased inflammation may provoke arrhythmias, since a role of pro-inflammatory macrophages in the pathogenesis of AF has recently been described [35,36]. Many of the characteristics of the atrial remodeling found in *JDP2 mice* resemble atrial remodeling in *human* AF, e.g., atrial myocyte hypertrophy, atrial dilatation, increased fibrosis, alterations in connexin expression and atrial conduction, and dysregulation of myocyte calcium handling [37]. Moreover, recent clinical data also suggest a link between inflammation and AF [38].

In adult *mice*, the atrial impairments were preceded by ventricular remodeling and dysfunction, which occurred already after one week of JDP2 overexpression [24]. Thus, the development of atrial defects may not be independent from ventricular defects. However, the finding in juvenile *mice* revealed that JDP2 overexpression alone causes atrial defects in the absence of ventricular impairments [23]. Thus, both animal models may be interesting subjects for the analysis of AF development. Interestingly, studies on *mice* with heart-specific overexpression of CREM-Ib∆CX resemble AF induction in *JDP2-overexpressing mice* [39,40]. However, *CREM-Ib∆CX overexpressing mice* do not present any ventricular phenotype. On the transcriptional level, both JDP2 and CREM-Ib∆CX act as inhibitors of transcription on CRE-promoter elements, but CREM-Ib∆CX lacks the chromatin remodeling function of JDP2, which may be the reason for the more pleiotropic effects of JDP2. Since in patients ventricular dysfunction is often a prerequisite of AF development [2,3], in depth analysis of causes and mechanisms of AF induction in adult *JDP2 mice* may reveal new therapeutic approaches in the treatment of AF.

## 6. Summary and Conclusions

In summary, studies from transgenic *mice* have implicated the transcriptional modulator JDP2 in the development of cardiac remodeling culminating in HF and AF. Patient data suggest JDP2 may be a marker for development and progression of HF. Thus, JDP2 emerges as a novel molecular player in cardiac remodeling in HF and AF.

## Figures and Tables

**Table 1:** Summary of available studies mentioned in this review that describe effects of JDP2 related to heart disease. Only main effects of JDP2 on ventricles or atria are listed. Arrows indicate up- or downregulation of JDP2 in the respective study. MI—myocardial infarction, HF—heart failure, PBMCs—peripheral blood mononuclear cells, CMs—cardiomyocytes, AF—atrial fibrillation, KO—knockout.

JDP2 Expression	Disease/Provocation	Cell Type	Species	Ventricular Effects	Atrial Effects	Reference
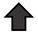	MI	PBMCs	*human*	predictor for HF development		[18]
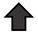	MI	PBMCs	*human*	central part in protein-protein network		[19]
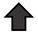	MI	PBMCs	*human*	target of hsa-mir-17-3p		[20]
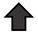	hypoxia	CMs	*human*			[22]
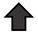	overexpression	CMs/heart	juvenile *mice*		atrial dilatation, AF	[23]
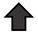	overexpression	CMs/heart	adult *mice*	ventricular dysfunction		[24]
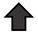	overexpression	CMs/heart	adult *mice*		atrial dilatation, AF	[25]
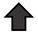	overexpression	isolated CMs	adult *mice*	contractile dysfunction, protection vs. hypertrophy and apoptosis		[26]
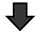	KO + pressure overload	global	*mice*	ventricular dysfunction		[27]
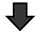	KO + *ATF3 KO* + pressure overload	global	*mice*	preserved ventricular function		[28]
